# 1-(4,5,6,7-Tetra­hydro­thieno[3,2-*c*]pyridin-5-yl)-2-{4-[3-(trifluoro­meth­yl)phen­yl]piperazin-1-yl}ethanone

**DOI:** 10.1107/S1600536812013591

**Published:** 2012-04-04

**Authors:** Shuang Zhi, Guo Zheng, Ying Liu, Deng-Ke Liu

**Affiliations:** a School of Environmental and Chemical Engineering, Tianjin Polytechnic University, Tianjin 300160, People’s Republic of China; b Tianjin Textile Fiber Interface Processing Technology Engineering Center, Tianjin Polytechnic University, Tianjin 300160, People’s Republic of China; c Tianjin Institute of Pharmaceutical Research, Tianjin 300193, People’s Republic of China

## Abstract

In the title mol­ecule, C_20_H_22_F_3_N_3_OS, the piperazine ring has a chair conformation, and the N—C(=O)—C—N torsion angle is −59.42 (14)°. In the crystal, weak C—H⋯O and C—H⋯π inter­actions link the mol­ecules into layers parallel to (101).

## Related literature

For details of the synthesis, see: Liu *et al.* (2008 ▶). For related structures, see: Niu *et al.* (2011 ▶); Zhi *et al.* (2011 ▶).
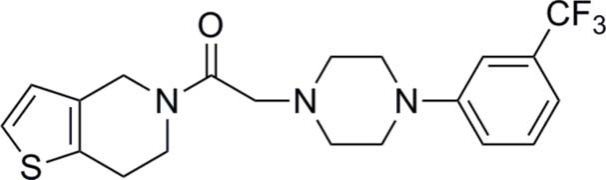


## Experimental

### 

#### Crystal data

C_20_H_22_F_3_N_3_OS
*M* *_r_* = 409.47
Monoclinic, 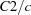

*a* = 32.692 (6) Å
*b* = 6.3772 (11) Å
*c* = 18.215 (3) Å
β = 92.985 (2)°
*V* = 3792.5 (11) Å^3^
*Z* = 8
Mo *K*α radiation
μ = 0.22 mm^−1^
*T* = 113 K
0.20 × 0.18 × 0.12 mm

#### Data collection

Rigaku Saturn724 CCD diffractometer
Absorption correction: multi-scan (*CrystalClear*; Rigaku/MSC, 2005 ▶) *T*_min_ = 0.958, *T*_max_ = 0.975
18550 measured reflections
4523 independent reflections
3449 reflections with *I* > 2σ(*I*)
*R*_int_ = 0.033

#### Refinement

*R*[*F* ^2^ > 2σ(*F* ^2^)] = 0.032
*wR*(*F* ^2^) = 0.097
*S* = 1.03
4523 reflections
253 parameters
H-atom parameters constrained
Δρ_max_ = 0.33 e Å^−3^
Δρ_min_ = −0.33 e Å^−3^

### 

Data collection: *CrystalClear* (Rigaku/MSC, 2005 ▶); cell refinement: *CrystalClear*; data reduction: *CrystalClear*; program(s) used to solve structure: *SHELXS97* (Sheldrick, 2008 ▶); program(s) used to refine structure: *SHELXL97* (Sheldrick, 2008 ▶); molecular graphics: *SHELXTL* (Sheldrick, 2008 ▶); software used to prepare material for publication: *CrystalStructure* (Rigaku/MSC, 2005 ▶).

## Supplementary Material

Crystal structure: contains datablock(s) global, I. DOI: 10.1107/S1600536812013591/cv5266sup1.cif

Structure factors: contains datablock(s) I. DOI: 10.1107/S1600536812013591/cv5266Isup2.hkl

Supplementary material file. DOI: 10.1107/S1600536812013591/cv5266Isup3.cdx

Supplementary material file. DOI: 10.1107/S1600536812013591/cv5266Isup4.cml

Additional supplementary materials:  crystallographic information; 3D view; checkCIF report

## Figures and Tables

**Table 1:** Hydrogen-bond geometry (Å, °) *Cg* is the centroid of the C14–C19 ring.

*D*—H⋯*A*	*D*—H	H⋯*A*	*D*⋯*A*	*D*—H⋯*A*
C6—H6*A*⋯O1^i^	0.99	2.56	3.4709 (17)	153
C13—H13*B*⋯O1^ii^	0.99	2.60	3.3607 (16)	133
C16—H16⋯*Cg*^iii^	0.95	2.61	3.3641 (13)	136

Symmetry codes: (i) 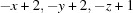; (ii) 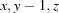; (iii) 
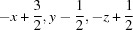
.

**Fig. 1.**
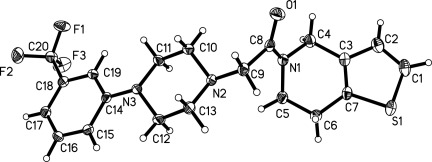
The molecular structure of (I)showing the atom-numbering scheme and 50% probability displacement ellipsoids.

## supplementary crystallographic information

### Comment

As a continuation of structural studies of thienopyridine derivatives (Niu *et al.*, 2011; Zhi *et al.*, 2011), we present here the title compound (I), which exhibits the antiplatelet aggregation ratio higher than ticlopidine.

In (I) (Fig. 1), pyridine ring with a half chair conformation is linked into the piperazine ring exhibiting a chair conformation by N1—C8—C9—N2 with a torsion angle of -59.42 (14)°. The dihedral angles formed between the thiophene plane(A), the phenyl ring (B) and the C10—C11—C12—C13 plane (C) are 29.48 (6)° (AB), 41.19 (7)° (AC) and 13.77 (8)° (BC), respectively. In the crystal structure, weak intermolecular C—H···O and C—H···π interactions (Table 1) link the molecules into layers parallel to (101).

### Experimental

Chloracetyl chloride was dropwised into the mixture of 4,5,6,7-tetrahydrothieno[3,2-*c*]pyridine, TEA and dichloromethane at 268 K. After stirring for 3 h, the solvent was evaporated and a light yellow oily substance was obtained by silica gel column chromatography. The light yellow oily substance then reacted with 1-(3-(trifluoromethyl) phenyl)piperazine in a mixture of acetonitrile and TEA. After stirring for 7 h at room temperature, the compound (I) was obtained by silica gel column chromatography (Liu *et al.*, 2008). Crystallization of the obtained yellow solid from acetone afforded light yellow crystals suitble for X-ray analysis.

### Refinement

The H atoms were positioned geometrically and refined using a riding model, with d(C—H)=0.95–0.99 Å, and *U*_iso_(H)=1.2*U*_eq_(C) of the parent atom.

### Crystal data

**Table:**

C_20_H_22_F_3_N_3_OS	*F*(000) = 1712
*M**_r_* = 409.47	*D*_x_ = 1.434 Mg m^−^^3^
Monoclinic, *C*2/*c*	Mo *K*α radiation, λ = 0.71073 Å
Hall symbol: -C 2yc	Cell parameters from 6174 reflections
*a* = 32.692 (6) Å	θ = 1.2–27.9°
*b* = 6.3772 (11) Å	µ = 0.22 mm^−^^1^
*c* = 18.215 (3) Å	*T* = 113 K
β = 92.985 (2)°	Prism, colourless
*V* = 3792.5 (11) Å^3^	0.20 × 0.18 × 0.12 mm
*Z* = 8	

### Data collection

**Table:**

Rigaku Saturn724 CCD diffractometer	4523 independent reflections
Radiation source: rotating anode	3449 reflections with *I* > 2σ(*I*)
Multilayer monochromator	*R*_int_ = 0.033
Detector resolution: 14.22 pixels mm^-1^	θ_max_ = 27.9°, θ_min_ = 1.3°
ω and φ scans	*h* = −42→40
Absorption correction: multi-scan (*CrystalClear*; Rigaku/MSC, 2005)	*k* = −8→8
*T*_min_ = 0.958, *T*_max_ = 0.975	*l* = −23→23
18550 measured reflections	

### Refinement

**Table:**

Refinement on *F*^2^	Primary atom site location: structure-invariant direct methods
Least-squares matrix: full	Secondary atom site location: difference Fourier map
*R*[*F*^2^ > 2σ(*F*^2^)] = 0.032	Hydrogen site location: inferred from neighbouring sites
*wR*(*F*^2^) = 0.097	H-atom parameters constrained
*S* = 1.03	*w* = 1/[σ^2^(*F*_o_^2^) + (0.0608*P*)^2^] where *P* = (*F*_o_^2^ + 2*F*_c_^2^)/3
4523 reflections	(Δ/σ)_max_ = 0.005
253 parameters	Δρ_max_ = 0.33 e Å^−^^3^
0 restraints	Δρ_min_ = −0.33 e Å^−^^3^

### Special details

**Table:**

Geometry. All e.s.d.'s (except the e.s.d. in the dihedral angle between two l.s. planes) are estimated using the full covariance matrix. The cell e.s.d.'s are taken into account individually in the estimation of e.s.d.'s in distances, angles and torsion angles; correlations between e.s.d.'s in cell parameters are only used when they are defined by crystal symmetry. An approximate (isotropic) treatment of cell e.s.d.'s is used for estimating e.s.d.'s involving l.s. planes.
Refinement. Refinement of *F*^2^ against ALL reflections. The weighted *R*-factor *wR* and goodness of fit *S* are based on *F*^2^, conventional *R*-factors *R* are based on *F*, with *F* set to zero for negative *F*^2^. The threshold expression of *F*^2^ > σ(*F*^2^) is used only for calculating *R*-factors(gt) *etc*. and is not relevant to the choice of reflections for refinement. *R*-factors based on *F*^2^ are statistically about twice as large as those based on *F*, and *R*- factors based on ALL data will be even larger.

### Fractional atomic coordinates and isotropic or equivalent isotropic displacement parameters (Å^2^)

**Table:**

	*x*	*y*	*z*	*U*_iso_*/*U*_eq_	
S1	1.129431 (9)	0.80354 (5)	0.452757 (18)	0.02545 (10)	
F1	0.76604 (3)	0.73309 (12)	0.07407 (4)	0.0338 (2)	
F2	0.72213 (2)	0.48600 (13)	0.05611 (5)	0.0396 (2)	
F3	0.78186 (3)	0.46014 (14)	0.01330 (4)	0.0394 (2)	
O1	0.97125 (3)	1.18330 (14)	0.40862 (6)	0.0298 (2)	
N1	1.00374 (3)	0.91401 (16)	0.35494 (6)	0.0198 (2)	
N2	0.92131 (3)	0.71911 (15)	0.37376 (6)	0.0174 (2)	
N3	0.84845 (3)	0.54625 (15)	0.30117 (5)	0.0170 (2)	
C1	1.14276 (4)	1.0434 (2)	0.41862 (8)	0.0293 (3)	
H1	1.1687	1.1077	0.4280	0.035*	
C2	1.11183 (4)	1.1299 (2)	0.37667 (7)	0.0266 (3)	
H2	1.1137	1.2619	0.3529	0.032*	
C3	1.07608 (3)	1.0008 (2)	0.37202 (7)	0.0206 (3)	
C4	1.03599 (4)	1.0538 (2)	0.33102 (7)	0.0238 (3)	
H4A	1.0286	1.2013	0.3408	0.029*	
H4B	1.0389	1.0378	0.2775	0.029*	
C5	1.01539 (4)	0.69138 (19)	0.35519 (7)	0.0215 (3)	
H5A	1.0255	0.6524	0.3067	0.026*	
H5B	0.9912	0.6036	0.3642	0.026*	
C6	1.04896 (4)	0.6520 (2)	0.41531 (7)	0.0221 (3)	
H6A	1.0372	0.6564	0.4643	0.026*	
H6B	1.0612	0.5118	0.4087	0.026*	
C7	1.08114 (3)	0.81855 (19)	0.41028 (7)	0.0198 (3)	
C8	0.97449 (4)	0.99396 (19)	0.39714 (7)	0.0194 (3)	
C9	0.94467 (4)	0.84037 (19)	0.42992 (7)	0.0201 (3)	
H9A	0.9602	0.7426	0.4632	0.024*	
H9B	0.9254	0.9193	0.4597	0.024*	
C10	0.89218 (4)	0.85104 (19)	0.33157 (7)	0.0207 (3)	
H10A	0.9069	0.9690	0.3095	0.025*	
H10B	0.8721	0.9103	0.3648	0.025*	
C11	0.86980 (4)	0.72588 (19)	0.27125 (7)	0.0212 (3)	
H11A	0.8498	0.8176	0.2442	0.025*	
H11B	0.8897	0.6758	0.2360	0.025*	
C12	0.87583 (4)	0.4201 (2)	0.35010 (7)	0.0240 (3)	
H12A	0.8962	0.3479	0.3206	0.029*	
H12B	0.8596	0.3116	0.3744	0.029*	
C13	0.89803 (4)	0.55328 (19)	0.40811 (7)	0.0232 (3)	
H13A	0.8779	0.6166	0.4404	0.028*	
H13B	0.9169	0.4642	0.4389	0.028*	
C14	0.82152 (3)	0.43532 (18)	0.25274 (6)	0.0163 (2)	
C15	0.80737 (3)	0.23339 (18)	0.26919 (7)	0.0183 (3)	
H15	0.8172	0.1666	0.3133	0.022*	
C16	0.77936 (3)	0.12972 (19)	0.22235 (7)	0.0192 (3)	
H16	0.7707	−0.0075	0.2347	0.023*	
C17	0.76363 (4)	0.22165 (18)	0.15800 (7)	0.0193 (3)	
H17	0.7441	0.1510	0.1264	0.023*	
C18	0.77749 (3)	0.42091 (18)	0.14144 (6)	0.0179 (2)	
C19	0.80626 (3)	0.52536 (18)	0.18650 (6)	0.0173 (2)	
H19	0.8158	0.6595	0.1724	0.021*	
C20	0.76198 (4)	0.5249 (2)	0.07173 (7)	0.0233 (3)	

### Atomic displacement parameters (Å^2^)

**Table:**

	*U*^11^	*U*^22^	*U*^33^	*U*^12^	*U*^13^	*U*^23^
S1	0.01591 (16)	0.0367 (2)	0.02358 (18)	0.00031 (13)	−0.00047 (12)	−0.00149 (14)
F1	0.0528 (5)	0.0192 (4)	0.0272 (4)	−0.0012 (3)	−0.0169 (4)	0.0034 (3)
F2	0.0294 (4)	0.0463 (5)	0.0411 (5)	−0.0079 (4)	−0.0182 (4)	0.0124 (4)
F3	0.0558 (5)	0.0456 (5)	0.0166 (4)	0.0109 (4)	0.0013 (4)	−0.0002 (4)
O1	0.0313 (5)	0.0203 (5)	0.0382 (6)	0.0007 (4)	0.0039 (4)	−0.0036 (4)
N1	0.0175 (5)	0.0211 (5)	0.0205 (5)	−0.0011 (4)	−0.0016 (4)	0.0002 (4)
N2	0.0159 (5)	0.0179 (5)	0.0180 (5)	−0.0004 (4)	−0.0035 (4)	0.0012 (4)
N3	0.0172 (5)	0.0158 (5)	0.0175 (5)	−0.0016 (4)	−0.0039 (4)	0.0039 (4)
C1	0.0185 (6)	0.0419 (8)	0.0278 (7)	−0.0089 (6)	0.0047 (5)	−0.0034 (6)
C2	0.0240 (6)	0.0319 (7)	0.0245 (7)	−0.0068 (6)	0.0067 (5)	−0.0006 (6)
C3	0.0184 (6)	0.0268 (6)	0.0166 (6)	−0.0019 (5)	0.0026 (5)	−0.0033 (5)
C4	0.0221 (6)	0.0272 (7)	0.0220 (6)	−0.0042 (5)	−0.0006 (5)	0.0030 (5)
C5	0.0198 (6)	0.0220 (6)	0.0224 (7)	0.0009 (5)	−0.0014 (5)	−0.0057 (5)
C6	0.0202 (6)	0.0205 (6)	0.0253 (7)	0.0019 (5)	−0.0014 (5)	−0.0020 (5)
C7	0.0160 (6)	0.0256 (6)	0.0176 (6)	0.0004 (5)	−0.0005 (5)	−0.0049 (5)
C8	0.0178 (6)	0.0220 (6)	0.0179 (6)	0.0004 (5)	−0.0056 (5)	−0.0009 (5)
C9	0.0190 (6)	0.0227 (6)	0.0184 (6)	−0.0001 (5)	−0.0016 (5)	−0.0022 (5)
C10	0.0221 (6)	0.0159 (6)	0.0236 (7)	−0.0005 (5)	−0.0046 (5)	0.0021 (5)
C11	0.0236 (6)	0.0178 (6)	0.0215 (6)	−0.0050 (5)	−0.0062 (5)	0.0054 (5)
C12	0.0275 (6)	0.0179 (6)	0.0256 (7)	−0.0021 (5)	−0.0096 (5)	0.0072 (5)
C13	0.0238 (6)	0.0232 (6)	0.0216 (6)	−0.0037 (5)	−0.0073 (5)	0.0066 (5)
C14	0.0135 (5)	0.0172 (6)	0.0181 (6)	0.0019 (4)	0.0015 (4)	0.0003 (5)
C15	0.0171 (5)	0.0179 (6)	0.0198 (6)	0.0012 (5)	0.0010 (5)	0.0028 (5)
C16	0.0187 (6)	0.0153 (6)	0.0240 (6)	−0.0010 (4)	0.0042 (5)	0.0007 (5)
C17	0.0185 (6)	0.0191 (6)	0.0201 (6)	−0.0006 (5)	−0.0003 (5)	−0.0043 (5)
C18	0.0190 (6)	0.0192 (6)	0.0156 (6)	0.0022 (4)	−0.0002 (5)	−0.0006 (5)
C19	0.0183 (5)	0.0152 (6)	0.0182 (6)	−0.0003 (4)	0.0003 (5)	0.0017 (5)
C20	0.0284 (7)	0.0209 (7)	0.0197 (6)	−0.0021 (5)	−0.0053 (5)	−0.0021 (5)

### Geometric parameters (Å, º)

**Table:**

S1—C1	1.7161 (14)	C6—H6A	0.9900
S1—C7	1.7241 (12)	C6—H6B	0.9900
F1—C20	1.3350 (14)	C8—C9	1.5254 (17)
F2—C20	1.3420 (14)	C9—H9A	0.9900
F3—C20	1.3407 (15)	C9—H9B	0.9900
O1—C8	1.2309 (15)	C10—C11	1.5153 (16)
N1—C8	1.3571 (15)	C10—H10A	0.9900
N1—C4	1.4644 (16)	C10—H10B	0.9900
N1—C5	1.4699 (16)	C11—H11A	0.9900
N2—C10	1.4591 (15)	C11—H11B	0.9900
N2—C13	1.4624 (15)	C12—C13	1.5114 (17)
N2—C9	1.4651 (15)	C12—H12A	0.9900
N3—C14	1.4047 (15)	C12—H12B	0.9900
N3—C11	1.4617 (15)	C13—H13A	0.9900
N3—C12	1.4695 (15)	C13—H13B	0.9900
C1—C2	1.3528 (19)	C14—C19	1.4041 (16)
C1—H1	0.9500	C14—C15	1.4059 (16)
C2—C3	1.4288 (17)	C15—C16	1.3865 (17)
C2—H2	0.9500	C15—H15	0.9500
C3—C7	1.3606 (18)	C16—C17	1.3854 (17)
C3—C4	1.5124 (17)	C16—H16	0.9500
C4—H4A	0.9900	C17—C18	1.3877 (17)
C4—H4B	0.9900	C17—H17	0.9500
C5—C6	1.5301 (17)	C18—C19	1.3860 (16)
C5—H5A	0.9900	C18—C20	1.4973 (17)
C5—H5B	0.9900	C19—H19	0.9500
C6—C7	1.5013 (17)		
			
C1—S1—C7	91.82 (6)	N2—C10—C11	110.82 (10)
C8—N1—C4	118.63 (11)	N2—C10—H10A	109.5
C8—N1—C5	123.45 (10)	C11—C10—H10A	109.5
C4—N1—C5	113.49 (10)	N2—C10—H10B	109.5
C10—N2—C13	107.61 (9)	C11—C10—H10B	109.5
C10—N2—C9	111.45 (9)	H10A—C10—H10B	108.1
C13—N2—C9	110.30 (10)	N3—C11—C10	111.36 (10)
C14—N3—C11	117.15 (10)	N3—C11—H11A	109.4
C14—N3—C12	116.58 (10)	C10—C11—H11A	109.4
C11—N3—C12	111.56 (9)	N3—C11—H11B	109.4
C2—C1—S1	111.82 (10)	C10—C11—H11B	109.4
C2—C1—H1	124.1	H11A—C11—H11B	108.0
S1—C1—H1	124.1	N3—C12—C13	111.82 (10)
C1—C2—C3	112.56 (12)	N3—C12—H12A	109.3
C1—C2—H2	123.7	C13—C12—H12A	109.3
C3—C2—H2	123.7	N3—C12—H12B	109.3
C7—C3—C2	112.61 (11)	C13—C12—H12B	109.3
C7—C3—C4	121.55 (11)	H12A—C12—H12B	107.9
C2—C3—C4	125.83 (12)	N2—C13—C12	110.42 (10)
N1—C4—C3	109.61 (10)	N2—C13—H13A	109.6
N1—C4—H4A	109.7	C12—C13—H13A	109.6
C3—C4—H4A	109.7	N2—C13—H13B	109.6
N1—C4—H4B	109.7	C12—C13—H13B	109.6
C3—C4—H4B	109.7	H13A—C13—H13B	108.1
H4A—C4—H4B	108.2	C19—C14—N3	121.12 (10)
N1—C5—C6	109.69 (10)	C19—C14—C15	116.85 (10)
N1—C5—H5A	109.7	N3—C14—C15	121.99 (10)
C6—C5—H5A	109.7	C16—C15—C14	121.31 (11)
N1—C5—H5B	109.7	C16—C15—H15	119.3
C6—C5—H5B	109.7	C14—C15—H15	119.3
H5A—C5—H5B	108.2	C17—C16—C15	121.54 (11)
C7—C6—C5	108.60 (11)	C17—C16—H16	119.2
C7—C6—H6A	110.0	C15—C16—H16	119.2
C5—C6—H6A	110.0	C16—C17—C18	117.41 (11)
C7—C6—H6B	110.0	C16—C17—H17	121.3
C5—C6—H6B	110.0	C18—C17—H17	121.3
H6A—C6—H6B	108.4	C19—C18—C17	122.05 (11)
C3—C7—C6	124.64 (11)	C19—C18—C20	118.61 (10)
C3—C7—S1	111.19 (9)	C17—C18—C20	119.32 (11)
C6—C7—S1	124.17 (10)	C18—C19—C14	120.80 (11)
O1—C8—N1	122.29 (12)	C18—C19—H19	119.6
O1—C8—C9	120.06 (11)	C14—C19—H19	119.6
N1—C8—C9	117.65 (11)	F1—C20—F3	106.27 (11)
N2—C9—C8	112.71 (10)	F1—C20—F2	106.51 (10)
N2—C9—H9A	109.0	F3—C20—F2	106.27 (10)
C8—C9—H9A	109.0	F1—C20—C18	112.62 (10)
N2—C9—H9B	109.0	F3—C20—C18	112.34 (10)
C8—C9—H9B	109.0	F2—C20—C18	112.35 (11)
H9A—C9—H9B	107.8		
			
C7—S1—C1—C2	−0.21 (11)	C14—N3—C11—C10	170.67 (10)
S1—C1—C2—C3	0.45 (15)	C12—N3—C11—C10	−51.32 (14)
C1—C2—C3—C7	−0.53 (17)	N2—C10—C11—N3	57.59 (13)
C1—C2—C3—C4	178.54 (12)	C14—N3—C12—C13	−170.23 (10)
C8—N1—C4—C3	108.44 (12)	C11—N3—C12—C13	51.50 (14)
C5—N1—C4—C3	−48.79 (14)	C10—N2—C13—C12	61.50 (13)
C7—C3—C4—N1	14.37 (17)	C9—N2—C13—C12	−176.74 (10)
C2—C3—C4—N1	−164.62 (12)	N3—C12—C13—N2	−57.23 (14)
C8—N1—C5—C6	−87.88 (13)	C11—N3—C14—C19	−18.70 (16)
C4—N1—C5—C6	68.10 (13)	C12—N3—C14—C19	−154.62 (11)
N1—C5—C6—C7	−46.98 (13)	C11—N3—C14—C15	163.73 (11)
C2—C3—C7—C6	−179.84 (11)	C12—N3—C14—C15	27.81 (16)
C4—C3—C7—C6	1.05 (19)	C19—C14—C15—C16	−0.63 (17)
C2—C3—C7—S1	0.36 (14)	N3—C14—C15—C16	177.05 (11)
C4—C3—C7—S1	−178.75 (10)	C14—C15—C16—C17	−1.02 (18)
C5—C6—C7—C3	15.39 (17)	C15—C16—C17—C18	1.02 (17)
C5—C6—C7—S1	−164.84 (9)	C16—C17—C18—C19	0.64 (17)
C1—S1—C7—C3	−0.09 (10)	C16—C17—C18—C20	178.81 (11)
C1—S1—C7—C6	−179.89 (11)	C17—C18—C19—C14	−2.33 (18)
C4—N1—C8—O1	8.89 (18)	C20—C18—C19—C14	179.49 (10)
C5—N1—C8—O1	163.72 (11)	N3—C14—C19—C18	−175.44 (10)
C4—N1—C8—C9	−171.93 (10)	C15—C14—C19—C18	2.25 (17)
C5—N1—C8—C9	−17.10 (16)	C19—C18—C20—F1	−23.30 (16)
C10—N2—C9—C8	−69.20 (13)	C17—C18—C20—F1	158.47 (11)
C13—N2—C9—C8	171.33 (9)	C19—C18—C20—F3	96.66 (13)
O1—C8—C9—N2	119.78 (12)	C17—C18—C20—F3	−81.57 (14)
N1—C8—C9—N2	−59.42 (14)	C19—C18—C20—F2	−143.58 (11)
C13—N2—C10—C11	−61.83 (13)	C17—C18—C20—F2	38.19 (16)
C9—N2—C10—C11	177.12 (10)		

### Hydrogen-bond geometry (Å, º)

Cg is the centroid of the C14–C19 ring.

**Table:**

*D*—H···*A*	*D*—H	H···*A*	*D*···*A*	*D*—H···*A*
C6—H6*A*···O1^i^	0.99	2.56	3.4709 (17)	153
C13—H13*B*···O1^ii^	0.99	2.60	3.3607 (16)	133
C16—H16···*Cg*^iii^	0.95	2.61	3.3641 (13)	136

Symmetry codes: (i) −*x*+2, −*y*+2, −*z*+1; (ii) *x*, *y*−1, *z*; (iii) −*x*+3/2, *y*−1/2, −*z*+1/2.
